# Colorectal carcinoma in the course of inflammatory bowel diseases

**DOI:** 10.1186/s13053-019-0118-4

**Published:** 2019-07-12

**Authors:** Andrzej Hnatyszyn, Szymon Hryhorowicz, Marta Kaczmarek-Ryś, Emilia Lis, Ryszard Słomski, Rodney J. Scott, Andrzej Pławski

**Affiliations:** 1 Health Care Center, Independent Public Hospital, Chałubińskiego 7, 67-100 Nowa Sól, Poland; 2 Institute of Human Genetics, Polish Academy of Sciences, Strzeszyńska 32, 60-479 Poznań, Poland; 3 Department of Biochemistry and Biotechnology, University of Life Sciences, Dojazd 11, 60-632 Poznań, Poland; 4 Division of Molecular Medicine, NSW Health Pathology (Newcastle) New South Wales, Newcastle, NSW 2308 Australia; 5 School of Biomedical Sciences, University of Newcastle, Newcastle, NSW 2308 Australia; 6 Department of General and Endocrine Surgery and Gastroenterological Oncology, Poznań University of Medical Sciences, Przybyszewskiego 49, 60-355 Poznań, Poland

**Keywords:** Colorectal cancer, Inflammation, Crohn disease, Ulcerative colitis, IBD, Inflammatory bowel disease

## Abstract

**Background:**

Colorectal cancer (CRC) and inflammatory bowel disease (IBD) are the most prevalent diseases of the digestive system, and their association is unequivocal. A long-standing inflammatory process is one of the causes of sporadic as well as inherited cancers as it impacts on malignant transformation in a wide variety of neoplastic diseases, including colorectal cancer.

**Methods:**

An extensive publication search was performed in Medline and PubMed database. The keywords: colorectal carcinoma, inflammation, Crohn disease, ulcerative colitis and inflammatory bowel disease were used.

**Results:**

The nucleotide-binding oligomerization domain-containing protein 2 (NOD2) and toll like receptor (TLR) signaling pathways are clearly involved in the inflammatory process and are therefore implicated in the transformation of normal colonic mucosa to premalignant and malignant disease. Focal sites of inflammation could significantly increase the risk of initiation and development of cancer. Altered inflammatory activity is likely to be a result of either a disturbance of intestinal bacterial flora or an inadequate cellular response to it. Additionally, increasing the level of inflammation-related factors may also interfere with the control of cellular proliferation.

**Conclusions:**

This review shows an overview of the genetic and environmental factors that appear to influence both the occurrence of IBD and CRC with particular reference to *NOD2* and *TLRs* as well as pro- and anti-inflammatory cytokines associated with tumor initiation and progression (encompassing both tumor invasion and metastases), as they constitute potential targets for therapeutic intervention.

## Background

Colorectal cancer (CRC) is the third most common malignancy and fourth leading cause of cancer-related mortality worldwide. There is a statistically significant association between countries affluence and the incidence of CRC. Developed countries account for greater than 55% of all CRC cases [1, 2]. Differences in the occurrence of CRC are over 10-fold: from the highest rates observed in Australia, New Zealand, Western Europe and the United States to the lowest in Africa and Central Asia [3, 4].

At present, over 75% of diagnosed CRC are classified as sporadic disease with no apparent first degree relatives affected with disease. Multiple environmental factors that include smoking, obesity and a diet rich in meat products and/or excessive alcohol consumption increases the risk of disease. CRC comprises a group of molecularly heterogeneous diseases, which are a result of different types of genomic, epigenomic, environmental and physiological factors that influence CRC development [4]. Most notably, an increased risk of developing CRC is associated with a variety of inherited disorders that include Familial adenomatous polyposis (FAP), Lynch syndrome, Peutz-Jegher’s syndrome and Juvenile Polyposis. In addition to these well-known inherited predispositions, chronic inflammation of the gastrointestinal tract is also tightly associated with a high risk of CRC [5–8]. We emphasize patients with inflammatory bowel diseases (IBD) and particularly those with ulcerative colitis (UC) represent a distinct entity burdened by an elevated risk of CRC. In this group of patients the incidence of CRC increases by 60% (i.e., colitis-associated colorectal cancer CAC) compared to that of the general population [9].

### Inflammatory bowel disease-related colorectal cancer

IBD constitutes a group of chronic, recurrent and incurable diseases of unknown etiology affecting the gastrointestinal tract that can ultimately lead to the destruction of normal intestinal architecture. The etio-pathogenesis of IBD is complex and multifactorial with a significnat influence associated with genetic susceptibility, immunological and environmental factors, which altogether interact and result in the dysregulation of the mucosal immune system of the gut leading to uncontrolled inflammation. Clinically, two main subtypes of IBD are defined: Crohn’s disease (CD), where inflammation may develop anywhere in the gastrointestinal tract from the mouth to the anus, and UC, where inflammation is limited to the colonic mucosa.

Patients with long-standing UC and CD are at a higher risk of developing CRC compared to the general population [9]. Although IBD-associated CRC accounts for approximately 2% of all CRC, the rate of death resulting from CRC in IBD patients ranges from 10 to 15% [10]. Population-based studies reveal that IBD and CRC have a similar prevalence worldwide with an increasing incidence in northwestern Europe and the USA. Furthermore, both diseases are associated with westernized behaviors and lifestyle [2].

The first meta-analysis performed by Eaden et al. in 2001 on the risk of IBD-associated CRC was based on the data of 54 478 patients from 116 studies, which revealed an overall CRC prevalence of 3.7% in UC patients. The incidence rate corresponded to a cumulative probability of 2% by 10 years; 8% by 20 years; and 18% by 30 years. The mean age of UC-associated cancer diagnosis was 43.2 years [11]. A nationwide Korean study involving 7061 UC patients reported comparable results with an estimated UC-associated CRC cumulative risk of 0.7% at 10 years, 7.9% at 20 years, and 33.2% at 30 years [12]. In contrast, a few population studies from Hungary, Sweden, United Kingdom and Denmark reported contradictory results with a cumulative risk of CRC in UC between 0 and 1.5% at 10 years, 1.1–5.4% at 20 years, and 2.1–10.8% at 30 years [13–16]. The meta-analysis performed by Canavan et al. based on 12 studies revealed the cumulative risk for patients with CD was 2.9% at 10 years, 5.6% at 20 years, and 8.3% at 30 years after a diagnosis of CD [17]. These results indicate the risk of developing CRC is similar for both CD and UC. Several subsequent reports from Caucasian populations revealed that CRC in the course of CD was characterized by much younger ages of cancer onset with tumors distributed evenly along the entire colon [18, 19].

A study by Watanabe et al. in Japan showed a decreased survival rate for patients with stage III UC-associated CRC than those with stage III sporadic CRC. In this study CRC associated with UC also tended to be multifocal and have a higher histological grade [20]. Jensen et al. reported similar findings from the Danish population, in which the overall mortality rate ratio of UC-associated CRC compared to sporadic CRC was 1.24 (95%CI: [1.02–1.51]) in the first year, and 1.17 (95%CI: [1.01–1.36]) after 5-years follow-up [21]. Furthermore, there are reports suggesting the risk of CRC is increased by at least 2-fold in IBD patients with a positive family history of CRC compared to patients without a family history of CRC [22, 23].

### Inflammation in colorectal cancer

The relationship between cancer and chronic inflammation was first described over 150 years ago by Rudolf Virchow (reviewed in [24, 25]). Since this hypothesis was put forward it has been confirmed by many clinical studies, but the pathogenesis and molecular biology of cancers associated with chronic inflammation remains the subject of much research. Chronic inflammation is observed in approximately 20% of all human cancers. The strongest correlation between long-standing inflammation and tumorigenesis is most apparent in CRC. [26]. Inflammation is one of the physiological factors that occurs as a protective response elicited by injury or the destruction of tissue. In effect, inflammatory-driven genetic alteration and epigenetic change occur, both important aspects of tumor initiation in CRC and particularly in CAC [27].

### Immune cells and cytokines in CRC

In IBD chronic inflammation is controlled by a complex interplay of innate and adaptive immune response mechanisms associated with disequilibrium in the production of Th1, Th2 and Th17 cytokines. This imbalance coupled with a defective immune response to pathogens promotes disease recurrence. The current concepts concerning circulating cytokines in IBD are still contradictory, but the cytokine profile in IBD tissue reveals that CD and UC are immunologically distinct [28]. Studies examining cytokines at the sites of inflammation in the colon has led to a paradigm in which UC is characterized by a predominance of cytokines related to T helper 2 (Th2) cells, whereas in the course of CD, Th1-associated cytokines prevail [29]. This is not reflected in the levels of systemic cytokines, many of which cannot be detected in the serum of IBD patients, mainly because they are restricted to the sites of inflammation or have relatively short half-lives. Further complicating this are significant issues in regards to the frequent extra-intestinal symptoms or comorbid diseases (such as stomatitis, skin inflammation, ocular manifestations, liver inflammation, rheumatoid arthritis and osteoporosis) that accompany IBD, reflecting the disruptive serum cytokine milieu [28, 30]. Cytokines secreted in response to infection and inflammation in the course of IBD may influence several stages of cancer initiation and progression. Tumour cells can react to host-derived cytokines that promote growth, attenuate apoptosis and facilitate invasion and metastasis. Furthermore, cytokine signaling is pleiotropic depending on the environment, one cytokine has the power to act on many different cell types to mediate diverse and sometimes opposing effects [31].

Cytokines such as TNF-α, IL-6 and IL-1 are involved in the promotion of CRC and CAC development whereas TGF-β and IL-10 are associated with an anti-inflammatory response. TNF-α is expressed in response to the initiation of inflammation and is secreted mainly by monocytes or macrophages. TNF-α maintains chronic inflammation and promotion of tumour progression and angiogenesis [32]. Activated TNF-α also triggers signaling pathways and transcription factors such as NF-κB. In turn, NF-κB plays a significant role in CRC and CAC tumorigenesis. Aberrant NF-κB activation occurs in 50% of CRC and CAC tumors [33]. Similarly to TNF-α, IL-1 acts as an initial alarm signal, which can activate a cascade of overlapping up-regulatory and pro-inflammatory responses. IL-1α and IL-1β are the most extensively studied cytokines within the IL-1 family.

Another pro-inflammatory cytokine is IL-6, which promotes tumor growth in human CRC cells. IL-6 appears critical for long-standing inflammation, where it is indispensable for the recruitment and activation of Th17 cells, and inhibits the function of regulatory T cells. IL-6 participates in the activation of signal transduction and activation of transcription 3 (STAT3). This trans-signaling pathway starts when IL-6 binds to the sIL-6R receptor on target cells, followed by dimerization of gp130 subunit and then activation of the JAK (Janus kinase). JAK induces recruitment and activation of STAT3, an oncogenic transcription factor involved in cell proliferation, survival, apoptosis and immune response [34].

Unlike pro-inflammatory cytokines, TGF-β together with IL-10 acts as an anti-inflammatory factor important in maintaining intestinal homeostasis and suppression of CRC tumorigenesis in early-stage cancer. However, these cytokines have a dual role during cancer development and may transform from an inhibitor to a promoter of disease [29]. Recently research has focussed on the use of manipulating cytokine signaling to create a treatment strategy that can protect or treat patients with chronic inflammation or CRC. The characteristic of cytokines acting on IBD and CRC is shown in Table 1 [35–43].

**Table 1:** The principal cytokines, playing a role in IBD and CRC [35–43]

Cytokine	Function in IBD	Function in CRC/CAC
Cytokines with pro-tumorigenic role		
IL-1β	Promotes IBD. Enhances the transcription and expression of IL-6 during the activation of cytokine cascade.	Mediates cancer-related inflammation and can be secreted by immune, stromal and tumor cells. Induces tumor cells proliferation, promotes the recruitment of myeloid-derived suppressor cells (MDSCs) to tumors, which supports cancer progression and induces the activation of the Wnt signaling pathway.
IL-4	Stimulates IgE class-switch recombination in B cells. Induces Th2 differentiation and IL-13 expression. Signals via STAT6.	May have distinct functions, depending on the tumor environment. Overexpress in early stages of CRC, appears to drive tumor development.
IL-6	Regulates inflammation by triggering a consolidated phase through monocyte recruitment.	Plays a formative role in the process of the chronic cancer-related inflammation, which becomes a genuine component of the tumor and the associated systemic reaction.
IL-8	Activates immune responses. Plays as a key mediator of immune tolerance.	Overexpressed in tumor tissue. Promotes tumor growth, metastasis and chemoresistance. Activates the Akt and MAPK pathways and promote the expression of genes responsible for cell proliferation, invasion and angiogenesis.
IL-17A	Necessary for intestinal homeostasis in IBD. Stabilizes epithelial barrier function. Induces pro-inflammatory cytokine/chemokine expression in endothelial cells; affect NF-κB-dependent signaling.	IL-17A serum levels are elevated in CRC patients. Positively correlates with tumor size or circulating tumor cells and poor survival. Indirectly activates STAT3 through IL-6. Activates the NF-κB pathway in CRC cells, thereby driving tumor survival and growth. Promotes the inflammation in CRC.
IL-22	Induces the production of antimicrobial peptides and mucus by epithelial cells. Drives intestinal inflammation.	Promotes tumor formation in bacterial driven colitis model. High levels of IL-22 in the serum or CRC tissue are predictive of a poor survival. Promotes resistance to chemotherapy. Promotes CRC development via induction of stemness in tumor cells.
IL-23	Suppress IL-10 in IBD intestinal mucosa and promotes chronic inflammation. Activates proliferation and cytokine expression in pathogenic Th17 cells; acts on STAT3-dependent signaling.	Induces the CRC. Overexpressed in primary CRC tissue. High IL-23 levels in primary tumor tissue are predictive of a higher rate of CRC metastasis. Indirectly promotes tumor cell survival.
IL-33	Promotes T_reg_-mediated control of colonic inflammation. Positively correlates with the extent of inflammation.	May promote or inhibit CRC development. Overexpressed in intestinal adenomas and adenocarcinomas in CRC patients. Indirectly affects the proliferation of tumor cells, decreasing the barrier function of the intestine, what allows for increased translocation of bacterial products to normally sterile tissues and induces the production of pro-tumorigenic cytokines, such as IL-6, by immune cells. Triggers the production of pro-angiogenic factors, which may promote CRC progression and metastasis.
TNF-α	Drives inflammation: regulates immune cell survival and function. Signals via NF-κB and MAPK pathways.	Links inflammation and cancer. In CRC, it is produced mainly by activated macrophages. Overexpressed in CRC tissues. TNF-α serum levels positively correlates with CRC progression and reduced patient survival. Promotes cell survival and CRC development. Contribution of TNF-α to CRC may be determined by the timing of its secretion during tumorigenesis or the type of the immune cells secreting it.
TGF-β	Inhibits cell growth and activation.	May function as a potent tumor suppressor that induces cell cycle arrest in early tumors, but high TGF-β levels in the primary tumor or serum correlates with poor survival. Promotes IL-11 secretion by CAFs, which in turn activates STAT3 and drives the proliferation of tumor cells.
Cytokines inhibiting CRC development		
IL-12	Stimulates T_h_1 differentiation and effector cytokine production (IFN-γ, TNF-α). Functions via STAT4-dependent signaling.	Plays a central role both for the induction and the expansion of T_h_1 responses as well as the activation of cytotoxic immune effectors, such as NK and CD8^+^ T cells. Activates and induces IFN-γ production, which limits tumor growth and metastasis. High preoperative IL-12p40 serum levels predicted a better survival in CRC patients.
IL-15	Activates NK cells development	Regulates anti-tumor cytotoxicity and modulates the inflammatory tumor microenvironment.
IL-17F	Blockade of IL-17F ameliorates the inflammation in colitis.	In contrast to IL-17A it does not lead to the activation of NF-κB in CRC cells.
IL-18	Activates the inflammation.	Restricts inflammation in the intestine by limiting the differentiation of T_h_17 cells and promoting the expression of effector molecules in T_reg_. Participates in the repair of the intestinal epithelium, possibly via IL-22. Promotes protective host immunity mediated by cytotoxic cells, including CD8^+^ T lymphocytes and NK cells.
NF-κB	Activation of NF-κB is strongly induced in the inflamed gut of IBD patients. Correlates with the severity of intestinal inflammation.	Activates the transcription of target genes that influence cellular proliferation, migration and inflammation. Cancer cells exhibit aberrant constitutive NF-kB activation, which is involved in multiple signaling cascades related to carcinogenesis.
Cytokines with unclear contribution to CRC		
IL-9	Impairs mucosal wound healing. Regulates epithelial cell proliferation and barrier function. Acts on STAT3/STAT1/MAPK-dependent signaling.	Possibly promotes lymphomagenesis but also may inhibit the tumor growth by promoting anti-tumor immunity.
IL-10	Inhibits antigen presenting cells and pro-inflammatory cytokine expression by immune cells. Activates STAT3-dependent signaling.	Binding to its receptor activates STAT1, STAT3, and STAT5. In CRC patient, IL-10 serum levels increase over time during tumor progression. High preoperative serum levels of IL-10 correlate with poor survival of CRC patients.
IL-21	Growth and differentiation factor for germinal center B cells. Induces the activation of JAK1, JAK3, and mainly STAT1/STAT3.	Upregulated in patients with ulcerative colitis-associated colon cancer. May impact on the polarization of the T helper cell response in CRC. Its expression in human tumor positively correlates with disease-free survival.
GM-CSF	Promotes antigen presentation and maintains T cell homeostasis.	Overexpressed in primary colon tumors. Activates the JAK-STAT, the MAPK, and the PI3K pathways, which results in cell survival and proliferation.

### Pathogenesis of IBD associated CRC

Carcinogenesis proceeds via a sequence of molecular alterations that result in a premalignant state culminating in genomic instability and the appearance of carcinoma. IBD-associated CRC appears quite similar to sporadic CRC with disease development comprising almost the same alterations as found in sporadic CRC. CAC is manifested in epithelial dysplasia and resulting from two types of instability: CIN (chromosomal instability) and MSI (microsatellite instability) [44, 45]. These modifications are observed in both pathways of carcinogenesis, but the timing and frequency of these alterations in epithelial cells are different that allow the distinction between IBD-CRC and sporadic CRC [46, 47]. The sequence of events in the development of sporadic cancer consists of a molecular cascade that includes the loss of adenomatous polyposis coli (APC) function, aneuploidy, methylation changes, microsatellite instability, activation of the *KRAS* gene and COX-2 enzyme, changes in *DCC/DPC4* genes and loss of p53 function [48]. The differences in IBD-CRC formation concern the loss of APC and p53 function. Loss of APC function typically occurs earlier in patients with sporadic CRC, whereas in patients with IBD it is less frequent and takes place later in the development of malignancy. By contrast, loss of p53 function occurs much earlier in IBD-associated carcinogenesis, compared to patients with sporadic CRC [45, 46, 49]. Mutations in *TP53* seem crucial for neoplasia initiation in IBD patients and are observed in 50–85% of CACs [48].

### NOD2 and TLRs signaling pathways in IBD and CRC

Kurzawski et al. first described the link between *NOD2* polymorphisms and the risk of CRC in 2004 [50]. Initially, studies confirmed DNA lesions constituting an base insertion at nucleotide position 3020, which was linked to CRC developing at older ages. Suchy et al. who analyzed sequence variants in genes engaged in inflammatory response, confirmed this finding and concluded that polymorphisms in genes involved in the inflammatory response might be genetic modifiers of the disease and influence CRC risk in older persons [51]. Interestingly, Freire et al. found a significantly higher incidence of *NOD2* mutations in patients with CRC diagnosed under 60 years of age (28.6% vs. 10.4%, *p* = 0.015) and in female patients (24.4% vs. 10.4%, *p* = 0.048) [52]. Askling et al. in a population-based cohort study of 19,876 individuals with UC or CD revealed that familial CRC was associated with a more than 2-fold risk of CRC and an increase in the absolute risk of CRC at 54 years of age from 3.8 to 6.9%. Patients with a first-degree relative diagnosed with CRC before 50 years of age had a higher relative risk (9.2, C.I. = 3.7–23.0) and the highest absolute risk (29%) of disease. However, they did not observe any association with familial IBD [53].

A large meta-analysis performed by Tian et al. summarized earlier reports and proved that R702W, G908R, and 3020insC variants in *NOD2* irrefutably contribute to CRC susceptibility in Caucasians [54]. The latest report by Samadder et al., on a 16-year long study following a group of 9505 patients with IBD, 101 of whom developed CRC, revealed that patients with IBD have a 3- to 5-fold increased risk of CRC, but more specifically, those with CRC in a first-degree relative had an almost 8-fold increase in disease risk. They emphasized that family history may act as a simple measure to identify individuals with IBD at highest risk for CRC and suggested the need for enhanced surveillance in this particular group of patients [55]. Recent in vitro studies performed by Udden et al. have shown that NOD2 activates the NF-κB and MAPK (mitogen-activated protein kinase) signaling axis in response to bacterial muramyl dipeptide (MDP) and inhibits TLR-mediated activation of these pathways and production of inflammatory mediators [56].

Toll-like receptors (TLRs) are mediators of inflammation in the gastrointestinal tract and may act as modulators of CRC risk. Activation of TLR signal transduction pathways induces numerous genes, including inflammatory cytokines, chemokines, and antigen presenting molecules. There are TLR variants that are associated with deregulation of their signaling function that are major contributing factors to the predisposition and susceptibility to IBD. [57].

Aberrant TLR signaling causes changes in commensal bacterial composition, intolerance and disturbance of mucosal homeostasis. Additionally, as sensors of cell death and tissue remodeling, TLRs have also been shown to be key receptors involved in inflammation-driven carcinogenesis. Several *TLRs* have been examined in the intestine, but the most important with respect to inflammation appear to be *TLR2* and *TLR4*, the expression of which has been shown to be increased in macrophages of IBD patients [58]. Slattery et al. examined 1555 individuals with CRC and 1956 controls for *TLR2*, *TLR3*, and *TLR4* with the risk of developing colon or rectal cancer. They showed that *TLR3* (rs3775292) and *TLR4 (*rs11536898) polymorphisms were significantly associated with rectal (although only weakly) and colon cancer, respectively. Although, they studied a group consisting of individuals with no previous history of CRC or any known (as indicated on the pathology report) diagnosis of FAP, UC or CD [59]. One of latest reports regarding *TLR*s variants in a group of patients with advanced and metastatic CRC indicated that *TLR4* Asp299Gly, *TLR4* Thr399Ile, TLR9 T1237C and T1486C and *TLR2–*196 to − 174 *del*/*del* homozygous genotypes were significantly associated with CRC. Moreover, *TLR4* Asp299Gly and Thr399Ile polymorphisms were significantly associated with concomitant *KRAS* gene mutations [60]. Both of these SNPs are located in the coding region of the *TLR4* ectodomain, which results in an amino acid substitution known to reduce cytokine expression, contributing to an increased susceptibility to CRC [61, 62].

Taken together, *NOD2* plays a critical role in the suppression of inflammation and tumorigenesis in the colon via down-regulation of the TLR signaling pathways. There remains a need for further research into families where IBD patients originate, especially those who carry polymorphic variants in genes involved in the inflammatory response and in particular, *NOD2* and the *TLR*s. A comprehensive study of *NOD2* and *TLR*s that include a thorough examination of genetic variance in these genes, differential expression levels and epigenetic modifications are crucial to understand normal and pathological function of these genes such that appropriate design and development of novel therapeutic approaches for CRC treatment can be undertaken.

### Epigenetic modifications in CRC

The CpG island hypermethylation in several gene promoters contributes to the pathogenesis of CRC resulting in gene silencing. Hypermethylation of the promoter region of a gene results in reduced gene expression and is associated with a failure to appropriately respond to the cellular environment. Two genes are most frequently affected by aberrant promoter methylation are *hMLH1* and *CDKN2A*. A loss of *MLH1* expression via epigenetic silencing is associated with microsatellite instability (MSI), observed in a proportion of both sporadic and IBD-associated CRCs [44, 63]. Silencing of *CDKN2A*results in aberrant signaling culminating in cellular proliferation [64].

Activation of cyclooxygenase enzyme COX-2 is also related to the pathogenesis of advanced CRC. Studies of COX-2 expression have shown it has elevated levels in various cancers and links the inflammatory response with CRC. Over-expression of COX-2 is induced by inflammation suggesting this is an early stage in tumour development. COX-2 contributes to increased proliferation and angiogenesis, both of which are correlated with tumour initiation and progression [44, 65]. Another key constituent of the pathogenic process is oxidative stress. Elevated levels of reactive oxygen and nitrogen species are associated with DNA damage thereby contributing to mutational load [44]. Active inflammation in IBD patients results in increased levels of inflammatory cells that include neutrophils and macrophages. These cells produce free radicals and pro-oxidant molecules called RONs (reactive oxygen and nitrogen forms) [48], which are linked to DNA and protein damage. Alterations in DNA or protein brought about by free radical attack can result in affecting essential growth-regulatory genes and may be implicated in IBD and CRC. DNA damage product concentration, namely the mutagen 8-hydroxydeoxyguanosine (8-OHdG) was reported to be increased in patients with IBD [66, 67], suggesting an etiological role in the development of malignancy in this disorder.

### Microbiota in CRC

It is well established that the intestinal microbiota plays a crucial role in the pathogenesis of CRC by altering intestinal bacterial biofilms, micro-environmental homeostasis, and immune reaction. Bacterial biofilms act as a first line of defense against invading microbes capable of inducing an inflammatory response and the generation of genotoxic compounds derived from bacteria. There is evidence to indicate that microbiotic imbalance contributes to the development of IBD-associated CRC by initiating and maintaining colonic mucosal inflammation [68, 69]. Abnormal bacterial microflora or disruption of homeostasis may promote inflammation in the large intestine, resulting in damage to the mucous membrane and subsequently, the development of neoplastic lesions. Pathogenic microorganisms contribute to intestinal inflammation through the activation of pattern recognition receptors (TLRs) or by endocytotic processes, toxin secretion, invasion or adherence [70].

TLRs are specific detectors of microbes and signal the immune system of any disequilibrium in microbial homeostasis, via the activation of transcription factors (i.e. NF-κB) and the secretion of cytokines. Research using mice that over-expressed TLR-4 showed increased inflammatory mediator levels: TNF-a, COX-2, and IL-12 and accordingly higher severity of both DSS (dextran sulfate sodium) induced colitis and CAC [71].

Gastro-intestinal inflammation may be the result of a disturbances in the ratio between tolerogenic and pro-inflammatory microflora that results in a breakdown of gut homeostasis and consequent mucosal inflammation. Changes in the number, diversity and stability of bacteria, in particular belonging to the *Clostridia* group, may lead to the development of intestinal diseases including CRC. The change of normal physiological into pathological processes, referred to as dysbiosis, may also affect CAC development. There is increasing evidence to suggest that the enterotoxic bacteria, *Bacteroides fragilis* and *Escherichia coli* promote the development of CRC. The presence of these bacteria is associated with tissue damage, STAT3 activation and IL-17 production resulting in colitis. Other pathogens that may contribute to the tumor progression are *Enterococcus faecalis* that activate the immune system via the production of reactive oxygen species (ROS). In turn *Streptococcus bovis* or *Bacteroides fragilis* are associated with the release of pro-survival and pro-angiogenic cytokines (i.e. IL-6, IL-8, IL-17) [27]. Furthermore, several studies carried out on IBD rodent models have revealed that commensal or specific bacteria are implicated in the development of IBD-associated CRC. *IL-10* knockout mice develop IBD as well as rectal dysplasia and adenocarcinoma induced by *Enterococcus faecalis* [72]. *IL-10* knock-out mice, under pathogen-free conditions, also developed colitis after the age of 3 months. CRC was observed in 25 and 60% of these mice after the ages of 3 and 6 months, respectively [73]. Moreover, modification of the enteric flora in *IL-10* knockout mice by probiotic lactobacilli was associated with a reduced prevalence of CRC and lower mucosal inflammatory activity [74]. There remains much still to be learned from studying gut microbiota and at present little is known about commensal bacteria and what role they play in IBD. At present, it appears that the key to a healthy gut is bacterial diversity rather than the presence of dominant bacterial strains.

## Conclusions

The association between inflammation and the initiation and development of cancer is unquestionable. Both processes are considered intertwined, as is observed in the context of cancer risk in patients with IBD. Chronic inflammation is not always recognizable at a clinical level and alone it may not be sufficient to trigger disease development. Notwithstanding, focal sites of inflammation could significantly increase the risk of initiation and development of cancer. Altered inflammatory activity is likely to be a result of either a disturbance of intestinal bacterial flora or an inadequate cellular response to it. Additionally, increasing the level of inflammation-related factors may also interfere with the control of cellular proliferation. A better understanding of the patho-mechanisms that drive inflammation in IBD patients is essential, as it is highly likely to result in the identification of additional therapeutic possibilities and reduce the risk of CRC in this population of patients.

## Data Availability

Not applicable.

## Abbreviations

8-OHdG: 8-hydroxydeoxyguanosine
APC: Adenomatous polyposis coli
CAC: Colitis*-*associated colorectal cancer
CD: Crohn’s disease
CIN: Chromosomal instability
COX-2: Cyclooxygenase 2
CRC: Colorectal cancer
DCC: Deleted in colon cancer gene
DPC4: Deleted pancreatic cancer gene
DSS: Dextran sulfate sodium
FAP: Familial adenomatous polyposis
hMLH1: human mutL homolog 1
IBD: Inflammatory bowel diseases
IL-1, IL-6 e.t.c.: Interleukin 1,6 e.t.c.
JAK: Janus kinase
MAPK: Mitogen-activated protein kinase
MDP: Muramyl dipeptide
MSI: Microsatellite instability
NF-κB: Nuclear Factor κB
NOD2: Nucleotide-binding oligomerization domain containing 2
p53: tumor protein p53
RONs: Reactive oxygen and nitrogen forms
ROS: Reactive oxygen species
STAT3: Signal transducer and activator of transcription 3
TGF-β: Transforming growth factor β
Th1, Th2,Th17: T helper (Th) cytokines
TLRs: Toll-like receptors
TNF-α: Tumor necrosis factor alpha
UC: Ulcerative colitis
